# Comparison of derived strain values of myocardial regions, levels, and segments by field strength and temporal resolution via cine bSSFP MR imaging

**DOI:** 10.1186/1532-429X-18-S1-Q16

**Published:** 2016-01-27

**Authors:** Eric J Keller, Peter M Smith, Benjamin Freed, Bradley D Allen, Bruce S Spottiswoode, Maria L Carr, Marie-Pierre Jolly, Kai Lin, James C Carr, Jeremy D Collins

**Affiliations:** 1 grid.465264.7Radiology, Northwestern University, Chicago, IL USA; 2 grid.465264.7Cardiology, Northwestern University, Chicago, IL USA; 3 Cardiovascular MR R&D, Siemens Healthcare, Chicago, IL USA; 4 grid.415886.6Medical Imaging Technologies, Siemens Healthcare, Princeton, NJ USA

## Background

Non-invasive assessment of myocardial strain has many promising implications in the early detection and management of cardiac disease. Preliminary work at our institution has shown deformation field analysis of balanced steady state free precision (bSSFP) cine MR images to compare favorably with speckle-tracking echocardiography. We have also illustrated that global strain values obtained by this MR-based technique demonstrate good agreement across field strengths and temporal resolutions in healthy volunteers. To further delineate the potential of this technique, we assessed strain values across field strengths and temporal resolutions for myocardial regions (endo-, meso-, and epicardium), levels (base, mid, and apex), and smaller divisions (segments) within each region and level.

## Methods

9 healthy volunteers (6 men, 44.3 ± 13.5 years) underwent imaging at 1.5T (MAGNETOM Aera, Siemens Healthcare GmbH, Erlangen) and 3.0T (MAGNETOM Skyra, Siemens Healthcare GmbH, Erlangen). Segmented bSSFP retrospectively ECG-gated cinegraphic imaging was performed and three short-axis slices acquired with temporal resolutions of 12.5 and 39.2 msec. Myocardial contouring in short axis views was generated via a previously described algorithm. Radial and circumferential strain values were then derived using an inverse consistent deformable registration algorithm on investigational prototype software. Peak and average peak strain values for myocardial regions, levels, and segments were compared across field strengths and temporal resolutions via two-tailed, paired t-tests.

## Results

Peak and average peak strain measurements showed good agreement (p > 0.05) across field strengths (1.5 and 3.0T) and temporal resolutions (12.5 and 39.2 msec) for radial and circumferential strain in all myocardial regions and levels (except circumferential strain in the epicardium). However, similar comparisons of smaller region and level segments often yielded significant differences (p < 0.05). Strain values varied significantly between myocardial regions and layers for the majority of field strengths and temporal resolutions (p < 0.05) similar to trends reported previously, e.g., the magnitude of circumferential strain increased on average from epicardium to endocardium and from base to apex (tables 1 & 2).

**Table 1:** p values for example peak strain data comparisons

Peak Strain	LV Circumferential			LV Radial		
Region	Endo	Meso	Epi	Endo	Meso	Epi
12.5 msec, 1.5T v. 3.0T	0.86	0.82	0.14	0.18	0.56	0.50
39.2 msec, 1.5T v. 3.0T	0.14	0.27	0.50	0.49	0.97	0.67
1.5T, 12.5 v. 39.2 msec	0.14	0.85	0.01**	0.20	0.88	0.45
3.0T, 12.5 v. 39.2 msec	0.75	0.21	0.06*	0.71	0.91	0.71
	Endo v. Meso	Meso v. Epi	Epi v. Endo	Endo v. Meso	Meso v. Epi	Epi v. Endo
12.5 msec, 1.5T	<0.01**	0.10*	<0.01**	0.02**	0.16	0.02**
12.5 msec, 3.0T	<0.01**	<0.01**	<0.01**	<0.01**	0.65	0.08*
39.2 msec, 1.5T	<0.01**	<0.01**	<0.01**	<0.01**	0.93	0.29
39.2 msec, 3.0T	<0.01**	<0.01**	<0.01**	0.04**	0.32	0.08*

**statistically significant difference < .05

*trend towards significance .05 - .10

**Table 2:** Average peak strain for myocardial regions and levels

Peak Strain	LV Circumferential			LV Radial		
Region	Endo	Meso	Epi	Endo	Meso	Epi
12.5 msec, 1.5T	-27.59	-24.36	-22.38	69.63	77.26	80.97
12.5 msec, 3.0T	-28.77	-25.03	-21.71	75.05	75.05	76.53
39.2 msec, 1.5T	-29.58	-24.62	-20.40	59.54	73.41	72.55
39.2 msec, 3.0T	-26.95	-23.05	-19.66	61.07	70.58	74.34
LV Level	Basal	Mid	Apical	Basal	Mid	Apical
12.5 msec, 1.5T	-16.34	-17.78	-16.21	37.56	45.65	27.18
12.5 msec, 3.0T	-15.98	-17.24	-18.63	32.44	42.13	33.88
39.2 msec, 1.5T	-16.02	-16.87	-20.39	37.07	42.05	35.75
39.2 msec, 3.0T	-14.26	-15.23	-18.08	27.47	32.45	25.97

## Conclusions

These results suggest deformation field analysis of bSSFP cine MR images is similar to speckle-tracking echocardiography in being sensitive to changes in global strain data for myocardial regions and levels but inconsistent for smaller areas of the myocardium. Although this technique has the advantage of enabling strain analysis of routine bSSFP cine sequences and avoiding limiting acquisition windows and operator-dependence seen with echocardiography, further development is needed to generate robust myocardial strain data at the segmental level.

